# Using Mendelian randomization to determine causal effects of maternal pregnancy (intrauterine) exposures on offspring outcomes: Sources of bias and methods for assessing them

**DOI:** 10.12688/wellcomeopenres.10567.1

**Published:** 2017-02-14

**Authors:** Deborah Lawlor, Rebecca Richmond, Nicole Warrington, George McMahon, George Davey Smith, Jack Bowden, David M Evans

**Affiliations:** 1 Medical Research Council Integrative Epidemiology Unit, University of Bristol, Bristol, UK; 2 School of Social and Community Medicine, University of Bristol, Bristol, UK; 3 University of Queensland Diamantina Institute, Translational Research Institute, Brisbane, Australia; 4 School of Women’s and Infants’ Health, The University of Western Australia, Perth, Australia

**Keywords:** Causality, developmental origins, intrauterine effects, Mendelian randomization, ALSPAC

## Abstract

Mendelian randomization (MR), the use of genetic variants as instrumental variables (IVs) to test causal effects, is increasingly used in aetiological epidemiology. Few of the methodological developments in MR have considered the specific situation of using genetic IVs to test the causal effect of exposures in pregnant women on postnatal offspring outcomes. In this paper, we describe specific ways in which the IV assumptions might be violated when MR is used to test such intrauterine effects. We highlight the importance of considering the extent to which there is overlap between genetic variants in offspring that influence their outcome with genetic variants used as IVs in their mothers. Where there is overlap, and particularly if it generates a strong association of maternal genetic IVs with offspring outcome via the offspring genotype, the exclusion restriction assumption of IV analyses will be violated. We recommend a set of analyses that ought to be considered when MR is used to address research questions concerned with intrauterine effects on post-natal offspring outcomes, and provide details of how these can be undertaken and interpreted. These additional analyses include the use of genetic data from offspring and fathers, examining associations using maternal non-transmitted alleles, and using simulated data in sensitivity analyses (for which we provide code). We explore the extent to which new methods that have been developed for exploring violation of the exclusion restriction assumption in the two-sample setting (MR-Egger and median based methods) might be used when exploring intrauterine effects in one-sample MR. We provide a list of recommendations that researchers should use when applying MR to test the effects of intrauterine exposures on postnatal offspring outcomes and use an illustrative example with real data to demonstrate how our recommendations can be applied and subsequent results appropriately interpreted.

## Introduction

The possibility that a wide-range of maternal pregnancy exposures, such as her gestational adiposity, smoking, diet and mental health, have long-term effects on an equally wide-range of post-natal offspring outcomes has gained such traction that it is influencing antenatal care. For example, the Independent Association of Diabetes and Pregnancy Study Groups (IADPSG) criteria for diagnosing gestational diabetes, which have been adopted by the World Health Organisation and many other national and international policy and guideline groups, aim to identify women whose children are at risk of future obesity, in order to ultimately prevent childhood obesity though antenatal care ^1^. However, evidence that the proxy measures used by IADPSG developers to indicate offspring obesity (high birthweight, birth skinfolds and cord-blood c-peptide) are accurate predictors of future risk of childhood obesity, or that treating women with the IADPSG criteria will effectively reduce childhood obesity, is lacking. In other areas women’s lifestyle in pregnancy is potentially being blamed for all future health risks in her offspring. It is essential, therefore, that methods are developed that can provide valid causal answers to questions about the long-term effects of intrauterine exposures. However, conventional methods are unlikely to be suitable; conventional approaches applied to cohort study data are likely to be influenced by residual confounding, and it is infeasible or extremely difficult to undertake randomised controlled trials (RCTs) of the effects of maternal pregnancy exposures on long-term offspring outcomes ^2^.

Mendelian randomization (MR), the use of genetic variants as instrumental variables (IVs) to test causal effects ^3– 5^, is increasingly used in aetiological epidemiology, including to test the effects of intrauterine exposures on long-term offspring outcomes ^6– 10^. Increased confidence in the use of MR to improve causal understanding has been achieved through proof-of-concept studies, such as those confirming the causal effects of greater body mass index (BMI) ^11^, systolic blood pressure ^12^, and low density lipoprotein cholesterol (LDLc) on coronary heart disease (CHD) ^13, 14^, and of smoking intensity and duration on lung cancer ^15– 17^, as well as concerted efforts by researchers using MR to acknowledge its underlying assumptions and how these might be violated, together with the development of methods to test assumption violations and/or be able to relax these ^3– 5, 18– 24^.

Methods to improve causal understanding of intrauterine exposures on offspring outcomes are important, given the likelihood for residual confounding in conventional multivariable analyses, and the infeasibility, or marked difficulty, of using RCTs to test effects of maternal pregnancy exposures on long-term offspring outcomes ^2^. The first paper to describe the MR method, highlighted the value of having genetic ( *MTHFR*) data on trios (both parents and offspring) when using MR to test the intrauterine effects of folic acid on offspring neural tube defects ^3^. However, the majority of methodological developments in MR have not considered the case, where maternal genetic variants are used as IVs to test the effect of a pregnancy (intrauterine) exposure on offspring outcomes. The aim of this paper is to describe specific ways in which the MR assumptions might be violated in studies concerning the effect of maternal pregnancy exposures on offspring outcomes, and to provide recommendations for how to test and potentially reduce the biases that might result from these assumption violations.

## Mendelian randomization assumptions and assessing causal intrauterine effects

**Figure 1.**
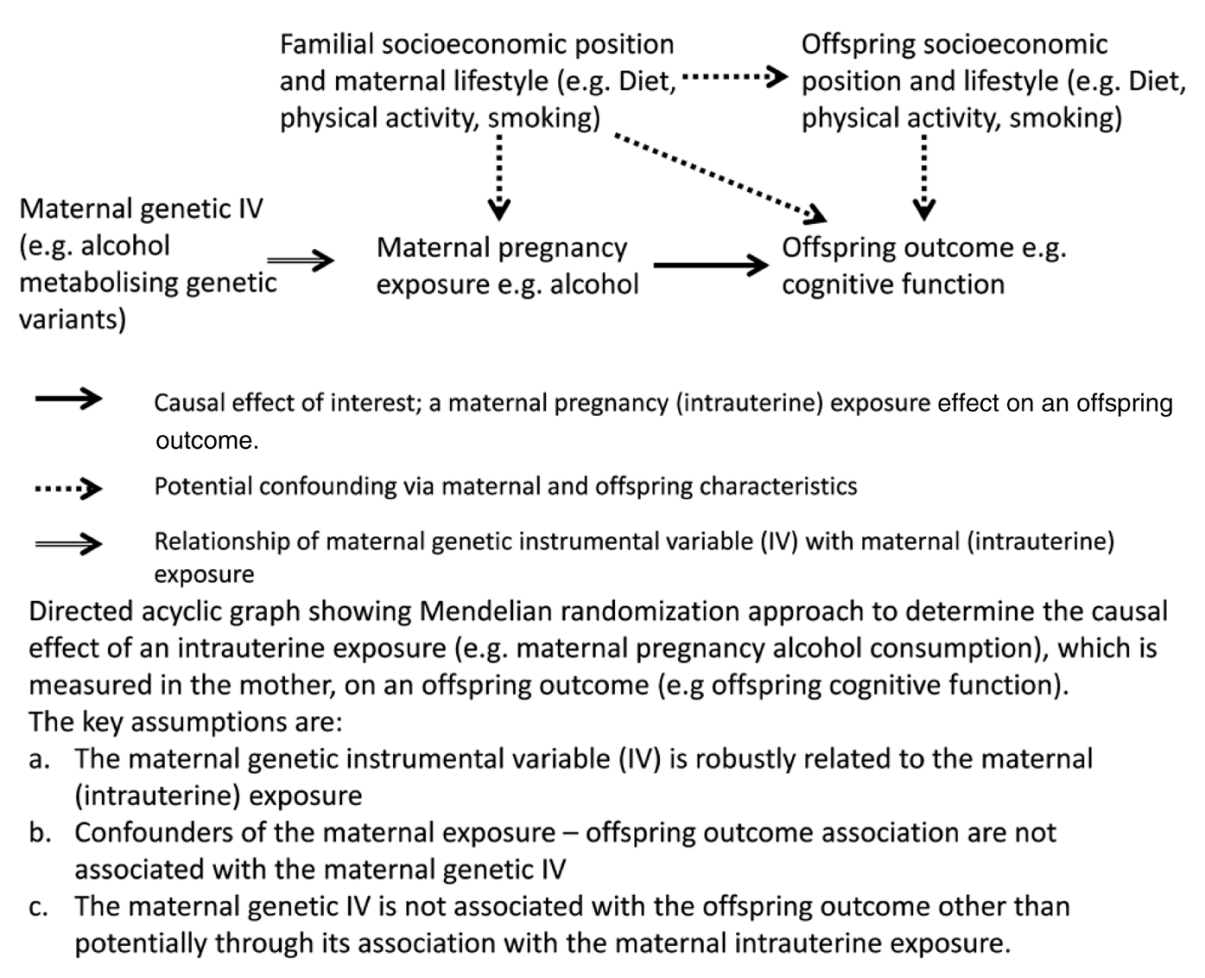
Directed Acyclic Graph of use of Mendelian randomization to assess developmental origins (intrauterine) causal effects on offspring outcomes.

Figure 1 shows the key IV assumptions as they apply to MR studies of maternal pregnancy exposures on offspring outcomes. We examine each of these in turn and discuss potential ways in which they may be violated.

### Maternal genetic instrumental variables are related to the maternal exposure during pregnancy

As with most MR studies, those that have been undertaken to test the effect of maternal pregnancy exposures on offspring outcomes have used variants that have been shown to be robustly related to the exposure in large genome-wide association studies (GWAS) conducted in non-pregnant women and men ^6, 10^. Some MR studies of maternal pregnancy effects, for example those related to alcohol consumption, have used candidate genes that are functionally related to the exposure of interest, but these have also been identified in non-pregnant women and men ^8, 9^. Thus, a key question for the use of MR in these studies is whether genetic variants identified in non-pregnant women and men are valid IVs for pregnancy exposures.

In theory, we might expect this to be the case given that genetic variants are determined at conception and many have been shown to relate to phenotypes across much of the life-course ^12, 25^. It has been shown that, for several genetic variants, associations with exposures measured in pregnancy are similar to those seen in GWAS of non-pregnant women and men. This appears to be so for BMI, fasting glucose, fasting lipids, 25(OH)D, adiponectin, thyroid levels and thyroid stimulating hormone ^10^. Genetic variants that have been shown to have genome-wide significant associations with type 2 diabetes have been shown to relate similarly to gestational diabetes ^10, 26– 28^, those related to smoking intensity and duration in GWAS, are related to difficulty quitting smoking in pregnancy ^29^, and variants known to influence alcohol metabolism relate similarly to alcohol consumption in pregnant women as they do in non-pregnant women and men ^30^. Whilst these findings are reassuring, it is important to demonstrate the relationship of genetic instrumental variables with the exposures of interest in pregnant women when using MR to test causal intrauterine effects on offspring outcomes ( Box 1).

Box 1. **Recommendations for using Mendelian randomization to determine causal effects of maternal pregnancy (intrauterine) exposures on offspring outcomes**

- Demonstrate a robust association of the maternal genetic instrumental variable (IV) with the exposure assessed in pregnancy.
- Explore the association of the maternal genetic IV with observed maternal and offspring potential confounders of the exposure-outcome association. Explore associations of the maternal genetic IV with potential confounders, with and without adjustment for the same offspring genetic variants that are going to be controlled for in the main MR analyses.
- Consider how likely genetic variants in the offspring, which are (strongly) associated with the offspring outcome of interest, overlap with genetic variants used in the maternal IV. Where the maternal exposure is the same as the offspring outcome, it is clear that there will be substantial overlap. In situations where the extent of any overlap is unclear, maternal genetic IVs should be looked-up in relevant publicly available datasets, such as those curated in MR-Base ^33^, to determine whether they are importantly related to the (offspring) outcome of interest.
  - Where there is no overlap between offspring genetic variants that influence their outcome and maternal genetic IV(s) there is no need to adjust for offspring genetic variants or undertake analyses with non-transmitted maternal genetic alleles.
  - If one is unsure about the extent to which there is overlap between offspring genetic variants that affect their outcome and maternal genetic IV(s), including after trying to explore this in publicly available datasets, it would be sensible to undertake MR analyses adjusted for offspring genetic variants and MR using non-transmitted maternal alleles as sensitivity analyses.
  - Where there is overlap between offspring genetic variants that affect their outcome and maternal genetic IV(s), the main MR analyses should use maternal genetic variant IVs adjusted for the same genetic variants in offspring (and ideally if possible also in fathers of the offspring) and/or MR using maternal non-transmitted alleles as IVs.
- Undertake as many of the following additional sensitivity analyses as possible with the available data:
  - Informed simulation analyses to test for acknowledged biases (e.g. due to violation of the exclusion restriction assumption via offspring and/or paternal genotype).
  - Weighted (using external weights) median method to explore violation of the exclusion restriction criteria, other than via offspring genotype. If using this in situations where there is concern about violation via offspring genotype, this sensitivity analysis is best done on IVs that are maternal genetic variants adjusted for the same offspring (and paternal if possible) genetic variants.
  - MR-Egger (using external weights) method to explore violation of the exclusion restriction criteria, other than via offspring genotype. If using this in situations where there is concern about violation via offspring genotype, this sensitivity analysis is best done on IVs that are maternal genetic variants adjusted for the same offspring (and paternal if possible) genetic variants.
- Triangulate with other approaches that can be used to test causal inference that make different assumptions to MR and have different sources of potential bias.

### Maternal genetic instrumental variables are not associated with potential confounders of the pregnancy exposure-offspring outcome association

There is empirical evidence that genetic variants are much less likely to be related to the wide-range of socioeconomic, lifestyle and associated phenotypic characteristics than these phenotypes are related to each other ^31^. However, we recommend that associations of maternal genetic IVs are checked with observed variables which are potential confounders of the maternal exposure and offspring outcome association. For some research questions, it will be optimal to adjust the maternal genetic IV for equivalent offspring (and paternal) genetic variants (see *Maternal genetic IV associations with offspring and paternal genetic variants*). Therefore, we recommend testing associations between maternal variants and observed potential confounders with and without adjustment for offspring (and paternal) genetic variants when such adjustments are used in the main MR analyses. Furthermore, as confounding paths between maternal pregnancy exposures and offspring outcome are likely to involve both maternal and offspring characteristics ( Figure 1), we recommend testing associations with relevant maternal and offspring observed confounders.

### Maternal genetic instrumental variables are only related to the offspring outcome through their relationship to the maternal pregnancy exposure (the exclusion restriction assumption)

**Figure 2.**
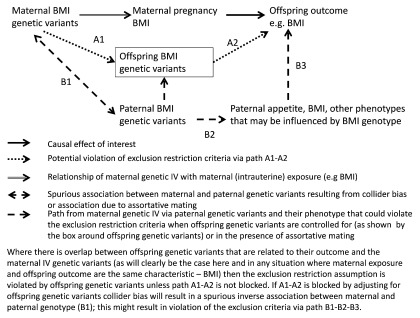
Directed Acyclic Graph illustrating the use of Mendelian randomization to assess developmental origins (intrauterine) causal effects on offspring outcomes, showing how the exclusion restriction criteria may be violated. BMI, body mass index; IV, instrumental variable.

***Maternal genetic IV associations with offspring and paternal genetic variants.*** A specific way in which the exclusion restriction assumption may be violated in studies of the effects of maternal pregnancy exposures on offspring outcomes, is via the offspring’s genetic variants ( Figure 2). The extent to which this will be a problem will depend on the extent to which any offspring genetic variants that are strongly related to their outcome are the same as variants used in their mother’s IV. This will almost definitely be a problem in the situation where the maternal exposure and offspring outcome are the same (or a very similar) characteristic (e.g. testing the causal effect of maternal pregnancy BMI on offspring BMI as in Figure 2). Yet, it is also likely to occur in some situations when the two are not the same. For example, some genetic variants related to BMI may have their effect on BMI via appetite control ^32^, metabolism or cardiorespiratory fitness. If an MR study were undertaken to test the causal effect of maternal pregnancy BMI on offspring appetite or cardiorespiratory fitness, it is plausible that there would be some overlap between maternal genetic IVs and offspring genetic variants, which are associated with these offspring outcomes. Thus, a key consideration is the extent to which the maternal genetic IVs overlap with offspring genetic variants that affect the offspring outcome of interest. It is increasingly possible to explore this using large publicly available datasets, such as those curated in MR-Base ^33^, in which the association of genetic variants being used in maternal IVs with the outcome of interest can be explored.

Where overlap between maternal genetic IVs and variants related to offspring outcomes are likely, the path from maternal genetic IVs to offspring outcomes via the offspring genotype needs to be blocked for the MR results to be valid. Consequently, many studies, which have used an MR approach to explore such relationships, have adjusted for offspring (fetal) genetic variants ^6, 7, 10^. However, this adjustment could introduce a spurious association between maternal and paternal genetic variants ( Figure 2). Intuitively, this is because once we adjust for offspring genetic variants we can, to some extent, predict paternal genetic variants given the genotype of the mother (e.g., if a mother is homozygous for a BMI increasing allele at a particular locus and her offspring is heterozygous at the same locus, then we know that father must be heterozygous or homozygous for the allele that is not associated with increased BMI). The spurious association that will be generated is inverse (i.e. maternal BMI increasing alleles are associated with paternal BMI decreasing alleles) because of the way that parental genotypes contribute to offspring genotype. This phenomenon is known as collider bias, because of its appearance in Directed Acyclic Graphs ( Figure 2) ^34– 36^. While there are many pregnancy/birth cohorts with genetic information on mothers and offspring, few have such data on fathers, and where they do this tends to be on a small selected sub-sample. Thus, it is rarely possible to be able to deal with this potential new bias by adjusting for paternal (as well as offspring) genetic variants. The issue then is whether it is better to adjust for offspring genetic variants (and risk introducing bias via paternal genetic variants and associated phenotype) or not (and risk introducing bias because of violating the exclusion restriction criteria via offspring genotype).

This is similar to what has been referred to as ‘M-bias’, where colliders exist and questions arise concerning whether or not to adjust for certain variable(s) ^36– 40^. Studies using simulation and real data suggest that in most such cases, the more proximal source of bias is likely to be the most important ^37– 40^. Thus, here we would anticipate that adjusting for offspring genetic variants, which are more proximal to maternal genetic variants, and offspring outcome, is more important than not, and that the potential bias from the more distal spurious association of paternal genetic variants with maternal variants is likely to be less problematic. In practice, whether the more proximal or distal bias is likely to be greatest will depend on the particular research question and the magnitudes of real and spurious associations.

For other questions, where maternal exposure is sufficiently different from the offspring outcome that it is unlikely that the same genetic variants that robustly influence maternal exposure would also influence (offspring) outcome, it might be reasonable to assume that there is no need to adjust for offspring genotype. For example, if we were interested in the causal effect of maternal pregnancy blood pressure on subsequent offspring depression, violation of the exclusion restriction assumption via offspring genotype is less likely than in the situation shown in Figure 2. This is because, whilst half maternal genetic variants for their blood pressure will also be transmitted to their offspring, offspring blood pressure variants (directly or via offspring blood pressure phenotype) are unlikely to have a (strong) association with offspring depression.

In addition to the spurious maternal-paternal genetic association induced by adjusting for offspring genetic variants, there may be a real association between maternal and paternal genetic variants generated by assortative mating. Assortment leads to an association between a couple’s genotypes and can be generated for a number of different reasons, including spouses being attracted to each other on the basis of heritable traits, including intelligence and physical appearance. This association (as with the potential spurious association of maternal-paternal genotype described above) could also result in bias, due to violation of the exclusion restriction criteria if there is assortment on the exposure of interest, and paternal genotype (unless it or offspring genotype, through which it would influence offspring outcome, are controlled for) and its associated phenotypes influence offspring outcome. Assortative mating would generally produce a positive bias. As adjusting for offspring genotype induces an inverse association between maternal and paternal genotype (and hence negative bias), it is possible that in the presence of assortative mating adjustment for offspring genotype produces a minimally biased result as the two cancel each other.

***Maternal genetic variants influence exposure pre- and post-pregnancy.*** Since genetic variants are allocated at conception, they conceivably influence traits across the entire life course. As a result, MR studies are often assessing the causal effect of a life-time cumulative exposure on outcomes ^12, 25, 41^. This is potentially problematic when we are using MR (or multivariable regression analyses) to address research questions concerned with exposure during a specific time period (such as the intrauterine period) ^41^.

**Figure 3.**
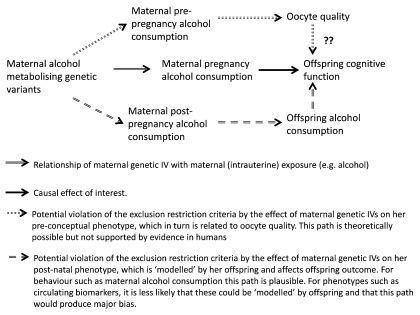
Directed Acyclic Graph illustrating use of Mendelian randomization to assess developmental origins (intrauterine) causal effects on offspring outcomes, showing illustrative examples of how the exclusion restriction criteria may be violated by pre-conceptual and post-natal maternal phenotype. IV, instrumental variable.

In MR studies of maternal pregnancy exposures on later offspring outcomes, it is theoretically possible that the exclusion restriction criteria is violated by a path from pre-pregnancy levels of the maternal exposure through an impact on primordial oocytes (the primary female gametes that are present in the female ovary from birth), which relates to subsequent offspring outcomes ( Figure 3). For example, studies in mouse models and non-human primates suggest that alcohol consumption in young adult females adversely affects oocyte quality and early stages of embryogenesis, even when alcohol consumption ceases before the completion of fertilisation and pregnancy ^42, 43^. Experimental evidence from animal models and on oocytes from women treated for infertility also suggest that maternal obesity may affect oocyte quality ^44^. If these effects are present in human females of reproductive age, the affected oocytes are capable of fertilisation and the changes to the oocytes impact on long-term offspring outcomes (all of which is currently is unclear), then this would provide a potential violation of the exclusion restriction criteria when exploring a ‘purely’ intrauterine effect via a pre-conceptual path ( Figure 3).

Where the (maternal) exposure of interest is a behaviour that is ‘visible’ to their offspring, such as alcohol consumption, there is also potential for violation of the exclusion restriction criteria by a postnatal path from maternal genotype to her postnatal exposure (e.g. alcohol consumption), and then via modelled offspring exposure to their outcome ( Figure 3). For maternal exposures that are less ‘visible’, for example circulating metabolite levels, postnatal effects are likely to be less problematic.

**Figure 4.**
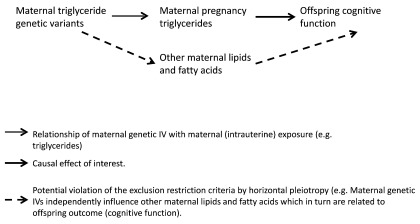
Directed Acyclic Graph illustrating use of Mendelian randomization to assess developmental origins (intrauterine) causal effects on offspring outcomes, showing how the exclusion restriction criteria may be violated by horizontal pleiotropy. IV, instrumental variable.

***True pleiotropic effects of the maternal genetic instrumental variable.*** The mechanisms described above in the two previous sections are specific to the situation where maternal genetic variants are used as instruments to test the effect of a maternal pregnancy (intrauterine) exposure on an offspring outcome. However, it is important to acknowledge that these studies are also (like all MR studies) vulnerable to violation of the exclusion restriction criteria, due to horizontal pleiotropy ( Figure 4) ^5, 21, 24^.

## Methods for assessing and limiting potential violations of the exclusion restriction criteria in MR studies of maternal pregnancy (intrauterine) effects

For many research questions concerned with intrauterine effects, violation of the exclusion restriction assumption via offspring genotype is likely to be a key concern. Therefore, researchers should start by considering the extent to which the maternal genetic IV variants overlap with offspring variants related to the outcome of interest. Where the maternal exposure is the same as the offspring outcome (e.g. testing the causal effect of maternal gestational BMI on postnatal offspring BMI), it is clear that the overlap is substantial. In situations where the extent of any overlap is unclear, maternal variants used as IVs should be looked-up in relevant publicly available datasets, such as those curated in MR-Base ^33^, to determine whether they are importantly related to the (offspring) outcome of interest.

In considering how the MR-Egger (see ***MR-Egger and median based methods to explore violations other than via offspring/paternal genotype***) method might work when addressing questions about intrauterine effects on offspring outcomes, we realised that it might be theoretically possible in some extremely specific cases that the problem of violation of the exclusion restriction assumption via offspring (and paternal) genotype might be solved by a very simple correction of the unadjusted MR result. However, we believe this would only work in a very specific situation where, amongst other criteria, the maternal exposure and offspring outcomes are exactly the same (e.g. testing the causal effect of maternal pregnancy BMI on adult offspring BMI), and where the model is additive and linear. If all criteria for this approach are present, a simple correction of the unadjusted MR result, achieved by subtracting 0.5 from this unadjusted result, this should yield an asymptotically unbiased estimate of the direct effect of maternal exposure on child’s outcome (i.e. assuming all the other core assumptions are fulfilled). The criteria required for this simple adjustment and our reasoning behind it are discussed in detail in Supplementary File 1: Section 1. However, this simple adjustment will be biased except in very specific situations and with very large sample sizes ( Supplementary File 1: Section 1 for details); therefore, we recommend that it is only used in sensitivity analyses, where it is plausible that all relevant criteria ( Supplementary File 1: Section 1) are met.

### Adjustment for offspring (and paternal) genotype

In situations where some genetic variants in the offspring, which relate to the offspring outcome, overlap with the maternal genetic variants used as IVs for maternal pregnancy exposure, we recommend adjusting for offspring genetic variants as part of the main MR analyses, and, if possible, simultaneously adjusting for paternal variants.

### Testing effects using maternal non-transmitted genetic variants

A second main analysis that we recommend (either as an alternative or additional analysis), in situations where we are concerned that there will be violation of the exclusion restriction criteria via offspring genotype, is to use a maternal genetic IV that is based only on her non-transmitted alleles ^45^. For each potential genetic instrument, if we can determine which of a mother’s alleles is not transmitted to their offspring, we can generate IVs that reflect the mother’s exposure, but are not related to offspring genotype (assuming the absence of assortative mating on the exposure) ^45^. If either or both mother or child are homozygotes then allele transmission can be unambiguously defined. To determine which allele has been transmitted from the mother in the situation where both mother and offspring are heterozygotes (for a bi-allelic variant) requires additional genetic information. For example, the most likely pattern of transmission can often be determined by constructing local haplotypes of adjacent SNPs in mother and offspring around each SNP instrument (assuming that such data is available, e.g. from genome-wide SNP arrays) ^45^.

### Sensitivity analyses using simulated data to explore violations due to offspring/paternal genotype

In addition to the methods described above – and in particular where it might not be possible to adjust for offspring (and/or paternal) genotype or construct haplotypes to use maternal non-transmitted alleles as IVs – we would suggest using simulations to explore the likely consequences of violation of the exclusion restriction criteria via offspring/paternal genotype. Here we provide methods, code and example results for doing such analyses.

***Methods for simulation analyses.*** We performed a number of simulations to quantify the bias using different approaches for estimating the causal relationship between maternal exposure and offspring outcome: (1) unadjusted MR analysis involving maternal genotype, maternal exposure and offspring outcome; (2) MR analysis involving maternal genotype, maternal exposure and offspring outcome adjusting for offspring genotype; (3) MR analysis involving maternal genotype, maternal exposure and offspring outcome adjusting for both paternal and offspring genotype; and (4) MR analysis utilizing the non-transmitted maternal alleles (we assumed that we knew the pattern of transmission precisely, which is effectively the case when dense genome-wide trio data are available), maternal exposure and offspring outcome.

For each simulation strategy, we tested the impact of different levels of effect of offspring and paternal genotype (via a direct path or via their phenotype) on offspring outcome (explaining 0, 0.5, 1, 2 or 5% of variation in offspring outcome). This range is to provide examples that might be seen across different plausible questions concerned with intrauterine effects on offspring outcomes ^46, 47^. We also allowed the IV strength, impact of confounders on offspring outcome, and the true causal effect of maternal exposure on offspring outcome to vary in these simulations. For each scenario, we generated 1000 replicates consisting of 10000 parent offspring trios. Full details of these simulations, including R code, are provided in Supplementary File 1: Sections 2 and 3 (R version 3.2.5 was used in these simulation studies).

***Results of simulation studies.*** Table 1 shows a selected number of illustrative examples from the large number (1800) of simulated scenarios. For all of these illustrative results the IV strength is the same (maternal genetic IV explaining 2% of the variation in maternal exposure), as is the net effect of confounders on the exposure and outcome (zero in all cases). We show results for a true non-null (positive) causal effect and for a null effect. The proportion of variance in the offspring outcome explained by offspring or paternal genotype (either directly or via the respective phenotypes) varied between 0 and 5%. The results show that for two approaches, adjustment for both offspring and paternal genotype and use of maternal non-transmitted genetic variants, all results are unbiased at any level of offspring or paternal genotype association with offspring outcome (similar results were obtained in the presence of non-genetic confounders; see full set of simulation results in Supplementary File 2– Supplementary File 7).

**Table 1.** Illustrative examples from simulation study results.

True causal effect ^a^	% variation offspring outcome explained by offspring genetics	% variation offspring outcome explained by paternal genetics	Difference in means of offspring outcome (in standard deviations units) per 1SD increase in maternal exposure that the IV is testing (SE)			
			No adjustment	Adjustment for offspring genetics only	Adjustment for offspring and father’s genetics	Using maternal non-transmitted alleles
0.10	0	0	0.10 (0.07)	0.10 (0.08)	0.10 (0.09)	0.10 (0.10)
0.10	0	1	0.10 (0.07)	-0.14 (0.08)	0.10 (0.08)	0.10 (0.10)
0.10	0	5	0.10 (0.07)	-0.42 (0.09)	0.10 (0.09)	0.10 (0.10)
0.10	1	0	0.46 (0.08)	0.10 (0.08)	0.10 (0.09)	0.10 (0.10)
0.10	1	1	0.45 (0.07)	-0.14 (0.08)	0.10 (0.09)	0.10 (0.10)
0.10	1	5	0.45 (0.08)	-0.43 (0.09)	0.10 (0.08)	0.10 (0.10)
0.10	5	0	0.90 (0.09)	0.10 (0.08)	0.10 (0.08)	0.09 (0.10)
0.10	5	1	0.90 (0.09)	-0.14 (0.08)	0.10 (0.08)	0.10 (0.10)
0.10	5	5	0.90 (0.09)	-0.43 (0.09)	0.10 (0.08)	0.10 (0.10)
0	0	0	0.00 (0.07)	0.00 (0.08)	0.00 (0.09)	0.00 (0.10)
0	0	1	0.00 (0.07)	-0.23 (0.09)	0.00 (0.09)	0.00 (0.10)
0	0	5	0.00 (0.07)	-0.52 (0.10)	0.00 (0.09)	0.00 (0.11)
0	1	0	0.35 (0.08)	0.00 (0.08)	-0.01 (0.09)	-0.01 (0.10)
0	1	1	0.35 (0.08)	-0.23 (0.08)	0.00 (0.09)	0.00 (0.10)
0	1	5	0.35 (0.08)	-0.52 (0.09)	0.00 (0.08)	0.00 (0.10)
0	5	0	0.79 (0.09)	0.00 (0.08)	-0.01 (0.08)	-0.01 (0.10)
0	5	1	0.79 (0.09)	-0.23 (0.08)	0.00 (0.08)	0.00 (0.10)
0	5	5	0.79 (0.09)	-0.53 (0.09)	0.00 (0.08)	-0.01 (0.10)

^a^Difference in mean offspring outcome in standard deviation (SD) units per 1 SD greater maternal exposure. In all simulations shown in this table the maternal genetic instrument explained 2% of the maternal exposure (R ^2^ = 0.02) and net confounding was zero. A full set of 1800 results from the simulation studies with a full range of different scenarios can be found in the Supplementary File 2– Supplementary File 7 (six separate excel files).

Except in the situation where offspring genotype was not associated with offspring outcome, failure to adjust for offspring genotype resulted in a large positive bias (exaggerating the true positive causal effect and producing a non-null positive effect when the true effect was null), which increased with stronger associations between offspring genotype and outcome. There was no impact on the causal effect estimate according to the magnitude of association between father’s genotype and offspring outcome, when no adjustment for offspring genotype is made. Adjustment for offspring genotype resulted in a modest negative bias (producing negative effects both when the true causal effect was positive and when it was null), unless the association between father’s genotype and offspring outcome was null. The magnitude of this negative bias increased with the strength of the association between father’s genotype and offspring outcome.

The results from all 1800 different simulation conditions that we undertook can be found in Supplementary File 2– Supplementary File 7 in six separate excel spreadsheets, each with four sheets. These are consistent with the general patterns highlighted in Table 1 and also show that confounders for the outcome (beyond offspring genotype) have relatively little impact on bias, except in the presence of weak instrument strength when they distort the true effect towards the direction of the confounding with greater bias as a result of greater magnitude of confounding. The power for each approach is also provided in these Supplementary files; the results confirmed the impact of instrument strength on power, and illustrate the slightly lower power of the maternal non-transmitted allele approach compared with other approaches.

### MR-Egger and median based methods to explore violations other than via offspring/paternal genotype

Two relatively novel methods for exploring the extent to which the exclusion restriction assumption might be violated are the MR-Egger and median based methods ^21^. Whilst these enable one to relax the exclusion restriction assumption, they have additional assumptions that are likely to be violated in situations where there is overlap between offspring genetic variants related to their outcome and the maternal genetic IVs. Therefore, we recommend these methods are used only as sensitivity analyses for exploring violations of the exclusion restriction assumption through paths other than offspring genotype (e.g. pre-conception or post-natal paths of horizontal pleiotropy), and are applied after adjustment for offspring (and ideally paternal) genotype. Methods for using MR-Egger or weighted medians with maternal non-transmitted alleles have not been developed, though as we discuss below it is theoretically possible that with large numbers of trios this approach could be combined with two-sample MR (see ***One- and two-sample Mendelian randomization***).

***MR-Egger.*** The MR-Egger method was developed to test for violation of the exclusion restriction assumption when using aggregate data from multiple genetic IVs ^21, 24^, as done in two-sample MR ^48^. It is related to the inverse-variance weighted (IVW) method, in which a weighted linear regression of each of the genetic IV–outcome coefficients on the genetic IV–exposure coefficients, with the latter orientated to be positive, is used. In IVW, the regression line is constrained to pass through zero (i.e. the exclusion restriction criteria is assumed and as such it is assumed if the genetic IV-exposure association is zero the genetic IV-outcome association must be zero) ^21^. Unlike the standard IVW approach that assumes no violation of the exclusion restriction criteria, the intercept in MR-Egger regression is not constrained to zero, and therefore provides an estimate of the average pleiotropic effect across all of the genetic variants ^21^. The MR-Egger method (through the MR-Egger slope) provides consistent estimates of the true casual effect, even if all genetic variants are invalid due to violation of the exclusion restriction assumption, but requires an additional assumption known as InSIDE (instrument strength independent of direct effect). The InSIDE assumption states that there should be no correlation between instrument strength (i.e. the SNP-exposure association) and the “direct” effect of the variant on the outcome (i.e. the effect on the outcome that is not mediated by the exposure of interest). The InSIDE assumption is likely to be violated in MR studies testing the effect of a maternal pregnancy exposure on an offspring outcome when the maternal exposure and offspring outcome are very similar ^21^. This is most easily illustrated if we think of the example where maternal exposure and offspring outcome are the same characteristic – e.g. BMI as in Figure 2. We know that the expected correlation between maternal genetic variants for BMI and offspring genetic variants for BMI at a single bi-allelic locus is 0.5 (since offspring inherit 50% of their genotype from each parent). Therefore, if the strength of association between maternal BMI genetic variants and maternal BMI is correlated with the strength of association between offspring BMI genetic variants and offspring BMI (as would be expected), then the InSIDE assumption will be violated (unless offspring genotype is controlled for). If offspring BMI is assessed in adulthood then this association is likely to be very high (i.e. approaching a correlation of 1), as the BMI variants will relate to BMI with the same magnitude in mothers and offspring. There is evidence that some genetic variants have age specific effects, such that their magnitude of association with BMI varies between infancy, childhood and adulthood ^49, 50^. Thus, if offspring BMI is measured in infancy or early childhood, the magnitude of a genetic BMI allele score with BMI may differ between pregnant adult women and their infant/childhood offspring. Nonetheless we would still anticipate some positive correlation between the two.

MR-Egger was developed for use within a two-sample MR framework using aggregate data, but studies of maternal pregnancy exposures on offspring outcomes will generally be done in the same sample(s) using individual participant data. The use of MR-Egger (and IVW) in one-sample MR is more problematic, however, because its performance deteriorates rapidly in the presence of weak instrument bias ^51, 52^. Unlike in the two sample context where simple methods exist to detect and adjust for weak instruments ^53^, no simple solution has yet been developed for a one-sample analysis (the first stage F-statistic alone is unlikely to be suitable for determining the extent of weak instrument bias when using MR-Egger or IVW). The direction of bias in this case is towards the (residually) confounded multivariable regression estimate rather than the null. This issue will be exacerbated if the genetic variants are weighted (by the magnitude of their effect on exposure) using internal weights (i.e. using the magnitudes of association with exposure in the same study in which the MR analyses are undertaken).

***Median based methods.*** Median based methods have also been developed to explore violation of the exclusion restriction criteria using two-sample MR with aggregate data. A simple (without weights) median method is obtained as the median of the ordered set of ratio estimates obtained from using each genetic variant as a single IV ^24^. The weighted median estimator is an extension of this method, which is more statistically efficient. It is the median of the inverse variance weighted empirical distribution function of IV estimates ^24^. This method can consistently estimate the true causal effect when at least 50% of the weight in the analysis stems from variants that are valid instruments. As a corollary, if a single genetic variant contributes more than 50% of the weight, then that variant must be valid. In MR studies where the maternal exposure and offspring outcome are the same (as in Figure 2) all of the variants are likely to be invalid.

Although the weighted median estimate will be biased towards the (residually) confounded multivariable regression estimate when using weak instruments in the one sample setting, the deterioration in performance is not expected to be as dramatic as in MR-Egger. This is because it is still a function of two-stage least squares (TSLS) estimates, rather than an (ordinary least squares) regression model.

## Illustrative example using real data

### Background

There is increasing concern that greater maternal adiposity in pregnancy results in greater offspring adiposity in later life via intrauterine mechanisms, and that this could perpetuate the obesity epidemic because female offspring born to women who are more adipose would enter their pregnancies more adipose and the effect would cycle through generations ^54, 55^. We have recently shown, using MR, that greater maternal pregnancy BMI is likely to be causally (via intrauterine mechanisms) related to offspring birthweight and ponderal index ^10^. We have also explored the likelihood that there may be lasting effects of maternal pregnancy BMI on offspring BMI and fat mass index (FMI) in offspring between ages 7 to 18 years ^6, 56^, using data from the Avon Longitudinal Study of Parents and Children (ALSPAC; http://www.alspac.bris.ac.uk) ^57, 58^. In this illustrative example, we examine the same research question and use ALSPAC data, but here we focus specifically on the analytical methods and sensitivity analyses recommended in Box 1. This example was chosen because it addresses an important public health question and also represents the case in which we might have most concern, since maternal exposure and offspring outcome are the same (BMI).

### Methods

Details of the study population and analyses that we used in this illustrative example are provided in Supplementary File 1: Section 4. We used two complementary approaches for our main MR analyses. First, we used a weighted allele score of 97 genetic variants that have been found to be robustly associated with BMI ^46^, along with adjustment for the same weighted allele score in offspring. Second, we examined the causal effect using the non-transmitted (to offspring) haplotype approach, as described by Zhang and colleagues ^45^. We also undertook three sensitivity analyses with real data - MR-Egger and weighted median methods applied to the maternal weighted allele score with adjustment for offspring allele and the simple subtraction of 0.5 from the unadjusted MR estimate and each value of its 95% confidence interval. In addition, we undertook simulation sensitivity analyses using the methods described in ‘ *Methods for simulation analyses’* and also in Supplementary File 1: Section 2. In the simulation analyses, we examined results assuming a true null result and also a true positive effect of 0.2SD change in the offspring BMI/FMI per 1SD change in maternal pregnancy BMI.

These statistical analyses were undertaken in StataIC version 14.

### Results

Each increase in weighted allele score in pregnant women in ALSPAC resulted in an average 0.026SD increase in their pre-pregnancy BMI and the allele score explained 2.6% of the variation in BMI; comparable results in the GWAS of men and (non-pregnant) women that were used to identify the 97 genetic instruments for this illustrative study, and to identify external weights, were 0.022SD and 2.7% ^46^. The first stage F-statistic was > 45. The maternal genetic IV (weighted allele score) was positively associated with household social class and to some extent with parity, but not notably with other potential confounders ( Supplementary File 1: Table S1).

The magnitudes of associations and patterns of differences between each approach were broadly the same for offspring BMI and offspring FMI. We found a positive effect of maternal early pregnancy BMI on offspring BMI and FMI at age 18 in the unadjusted MR weighted allele score using TSLS, which was considerably stronger than the multivariable regression result. However, with adjustment for offspring allele score (our first main analyses) this attenuated considerably and was consistent with the null ( Table 2). Similarly, when maternal transmitted alleles were used as the IV there was a strong positive effect (reflecting the transmission of BMI increasing alleles from mother to offspring and the effect of these on offspring BMI/FMI), but with the non-transmitted allele (our second main analyses) the point estimate was weakly negative and consistent with the null ( Table 2). For both BMI and FMI there was statistical evidence that the effects that both of our main MR methods were statistically inconsistent with the association from multivariable regression analyses (all p-values < 0.03).

**Table 2.** Mendelian randomization analyses using different approaches to assess the intrauterine effect of maternal pre-pregnancy body mass index on offspring body mass index (BMI) and fat mass index (FMI) at age 18 years.

MR method ^a^	N	Difference in mean BMI (SD) per 1SD greater maternal BMI (95%CI)	N	Difference in mean FMI (SD) per 1SD greater maternal BMI (95%CI)
*Main analyses*				
*Unadjusted maternal weighted allele* *score*	*2493*	*0.60 (0.37, 0.82)*	*2404*	*0.54 (0.31, 0.77)*
Maternal weighted allele score adjusted for same offspring weighted allele score	2493	0.01 (-0.23, 0.25)	2404	-0.01 (-0.26, 0.24)
*Maternal transmitted haplotype score*	*2482*	*1.36 (0.85, 1.86)*	*2393*	*1.27 (0.78, 1.77)*
Maternal non-transmitted haplotype score	2482	-0.04 (-0.36, 0.28)	2393	-0.07 (-0.40, 0.27)
*Sensitivity analyses*				
Inverse-variance weighted method with offspring adjusted IV ^b^	2493	0.11 (-0.02, 0.24)	2404	0.12 (-0.01, 0.25)
MR-Egger slope with offspring adjusted IV ^b^	2493	0.03 (-0.17, 0.23)	2404	0.11 (-0.09, 0.31)
MR-egger intercept with offspring adjusted IV ^b^	2493	0.006 (-0.006, 0.018)	2404	0.000 (-0.012, 0.013)
Weighted Median with offspring adjusted IV ^b^	2493	0.27 (0.03, 0.50)	2404	0.20 (-0.04, 0.44)
Unadjusted maternal weighted allele score minus 0.5	2493	0.10 (-0.13, 0.32)	2404	0.04 (-0.19, 0.27)
*Simulation sensitivity analyses* ^c^				
Assuming null: With no adjustment	10000	0.49 (0.33, 0.65)	10000	0.49 (0.33, 0.65)
Assuming null: Adjusted for offspring genotype	10000	-0.24 (-0.42, -0.06)	10000	-0.24 (-0.42, -0.06)
Assuming null: Adjusted for offspring and paternal genotype	10000	0.00 (-0.18, 0.18)	10000	0.00 (-0.18, 0.18)
Assuming null: Using maternal non- transmitted alleles	10000	0.00 (-0.22, 0.22)	10000	0.00 (-0.22, 0.22)
Assuming 0.1: With no adjustment	10000	0.60 (0.44, 0.76)	10000	0.60 (0.44, 0.76)
Assuming 0.1: Adjusted for offspring genotype	10000	-0.14 (-0.30. 0.02)	10000	-0.14 (-0.30. 0.02)
Assuming 0.1: Adjusted for offspring and paternal genotype	10000	0.10 (-0.12, 0.32)	10000	0.10 (-0.12, 0.32)
Assuming 0.1: Using maternal non- transmitted alleles	10000	0.10 (-0.10, 0.30)	10000	0.10 (-0.10, 0.30)
*Multivariable regression results for comparison* ^d^				
	1798	0.33 (0.28, 0.37)	1739	0.32 (0.27, 0.37)

^a^All Mendelian randomization (MR) methods use maternal 97 SNPs from Locke *et al.* GWAS ^46^. The main analyses are the maternal weighted allele score adjusted for offspring weighed allele score and the maternal non-transmitted analyses; the italicised unadjusted maternal weighted allele score and the transmitted maternal alleles (show italicised) are for comparison. The two stage least squares (TSLS) IV method was used in all four of these analyses. External weights from the recent GWAS ^46^ were used for the main analyses and inverse-variance weighted, MR-Egger and weighted median sensitivity analyses. ^b^These methods were applied to the maternal BMI genetic IVs (97 SNPs) adjusted for the same 97 BMI SNPs ^c^These show the results we might have expected to get for each method if the true result was 0 (null) or a 0.1SD increase in offspring BMI per 1SD increase of maternal pregnancy BMI. For all of these results based on simulated data maternal BMI allele score (instrument) is assumed to explain 2% of the maternal exposure (R ^2^ = 0.02), as is the offspring BMI allele score with their BMI, paternal allele score is assumed to explain 1% of variation in offspring BMI and we assumed the net confounding was zero. ^d^For comparison these are the multivariable regression results with control for household social class, maternal and paternal education, maternal smoking, offspring smoking and offspring sex and age at outcome assessment.

The MR-Egger intercept was consistent with the null and the slope was similar to the two main analysis results, suggesting no strong evidence for a causal intrauterine effect of maternal pregnancy BMI on offspring BMI or FMI at age 18-years ( Table 2). Both the IVW results and results obtained with a simple subtractions of 0.5 from the unadjusted MR results were consistent with the null but had point estimates that were positive and stronger that either the main result or the MR-Egger slope (though both were ~50% of the magnitude of the multivariable regression results). By contrast, the positive weighted median point estimate was of a similar magnitude to that of the multivariable regression result.

The simulation studies suggest that whether we assume a null, or positive, association the MR method with adjustment for offspring and paternal genetic variants and that using maternal non-transmitted alleles were unbiased. The simulated results with adjustment for offspring genetic variants only (i.e. without paternal adjustment) were modestly inversely biased, whereas this approach with real ALSPAC data (one of our main MR approaches) had point estimates very close to the null for both BMI and FMI. The difference between this simulated result and our real data may be explained by our assumption in the simulated data that paternal genotype will explain 1% of offspring BMI (after adjustment for offspring BMI – i.e. removing the path from paternal genotype to offspring BMI that would go through offspring genotype); in the ALSPAC cohort paternal genotype might have less impact on offspring BMI once offspring genotype is adjusted for.

### Discussion

We acknowledge that we have limited statistical power from this one study. However, the aim was to illustrate the series of analyses we recommend in Box 1, using a real example. The two main analyses – maternal genetic instruments adjusted for the same genetic variants in offspring and use of maternal non-transmitted variants as instruments – were consistent in suggesting that there was no strong causal intrauterine effect of maternal pregnancy BMI on offspring BMI and FMI at age 18. Our sensitivity analyses - MR-Egger slope, weighted median and subtraction of 0.5 from the unadjusted IV results – were also consistent with a null effect. Given the small sample size, we had anticipated that weak instrument bias might bias results from IVW, MR-Egger and the weighted median approaches towards the (residually) confounded multivariable results, with the extent of this bias being expected to be greatest for MR-Egger and least for the weighted median. We actually see the opposite, with little evidence of such bias for MR-Egger and greatest with the weighted median method. In ALSPAC, we only have genetic data on a small number of select fathers and were therefore unable to explore adjustment for offspring and paternal genotype, or use trios for determining maternal non-transmitted alleles. Overall the results suggest pregnancy BMI is unlikely to have a strong effect on offspring BMI, but we would want to explore this in additional larger studies.

## Conclusions and additional comment

The main output of this paper is a list of recommendations for undertaking MR in situations where one is interested in the causal effect of an intrauterine exposure that is measured and instrumented for in pregnant women on an outcome that occurs (and hence is measured) in their offspring. We have tried to provide a clear rationale for these recommendations but we do not claim that they are necessarily complete. As MR is further used to address such questions we envisage additional issues may be identified and also that additional methods (to those discussed here) will be developed. Below we discuss two further related issues to addressing questions of causal effects of maternal pregnancy exposures on offspring outcomes.

### One- and two-sample Mendelian randomization

In one-sample MR, both the genetic IV-exposure and genetic IV-outcome associations are computed in the same study sample; in two-sample MR the two are computed in independent samples ^48^. There are advantages to two-sample MR, in particular with increasing availability of complete summary results data from a large number of GWAS, it is possible to apply two-sample MR to these summary data and undertake MR in very large numbers of participants ^48, 59^. However, because it is essential to link exposure in mothers to outcome in their offspring it is not possible to use such summary data from two-samples to assess most questions related to intrauterine effects on offspring postnatal outcomes, though two-sample MR has been used to explore the causal effect of birthweight (as a proxy for some aspects of intrauterine environment) on later outcomes within the same individuals ^60– 62^. In theory two-sample MR could be used to explore the effect of maternal pregnancy exposures on offspring outcomes, but in general it would be essential to have individual participant data on maternal genetic variants that were used as IVs linked to offspring outcomes. For example, the association of maternal genetic instruments with offspring outcomes [ideally adjusted for offspring (and paternal) genotype] could be divided by results of the maternal genetic instruments with maternal exposure, with the latter obtained from a different sample to the former. Ideally, one would want the latter to assess maternal exposure during pregnancy, but if one were able to show (in a reasonably sized sample of pregnant women) that the genetic instruments related to maternal pregnancy exposure with the same magnitude and direction as that seen in the general (aggregate) GWAS data of (non-pregnant) women and men, it might be valid to use aggregate GWAS data for the genetic IV-‘maternal’ exposure sample (ratio estimate denominator). In practice, overlapping sample MR ^48^ might be more common and useful. For example, for expensive or unusual pregnancy exposures there may only be measurements in a sub-sample of the cohorts being used, in which case the denominator of the ratio IV estimate (i.e. association of maternal genetic IVs with pregnancy exposure) might be done only in that smaller sample, but the numerator (association of maternal genetic IVs with offspring outcome) done in the large sample.

The one exception to this general need to have maternal genetic IVs directly linked to offspring outcomes, would be in the case where it was possible to have ‘fully’ estimated maternal non-transmitted alleles. For example, if it were possible to have large numbers of trios with genetic data together with pregnancy exposure data from the mothers in those trios. Theoretically one could correctly identify the non-transmitted haplotype scores in the mothers and use those to determine the associations of these haplotype scores with the maternal pregnancy exposure in the sample from which the trios came. Then, look up the haplotype scores, which are the same as the maternal non-transmitted scores, in a general population sample to estimate their associations on ‘offspring’ outcomes. Currently, there are too few birth/pregnancy cohorts with genetic data on trios and maternal (potential exposure) measurements in pregnancy. However, as more pregnancy/birth cohorts collect genetic data on trois and work together collaboratively, there is future potential to explore this theoretical approach further.

### Combining results with other approaches in a triangulation framework

This paper highlights specific sources of bias when MR is used to explore the causal effect of maternal pregnancy exposures on offspring outcomes. Given the difficulty of establishing causality using conventional multivariable approaches or RCTs to address research questions concerned with the effect of maternal pregnancy exposures on long-term offspring outcomes, together with increasing pressure to direct antenatal care and public health policy towards preventing future ill-health via antenatal interventions ^54^, we feel that using methods such as MR is important in this field, and in Box 1 we provide recommendations for its use. We would also suggest that results from such MR studies are integrated with other approaches that a priori are assumed to have different key sources of bias in a triangulation framework ^41^. The idea of triangulation is that if the results from two or more different approaches, each of which has different and unrelated sources of bias, point in the same direction this supports those results pointing to the correct causal answer. In the case of the illustrative example that we present here, the fact that negative control studies (using paternal BMI as a negative control) ^63^, and within sibship analyses ^64^, each of which have different key sources of bias to the MR approach used here ^41^, all point towards there being no strong causal effect of maternal greater pregnancy BMI on her offspring BMI, strengthens the conclusion that there is no causal intrauterine effect in this specific example.

## Data availability

ALSPAC data used for this submission will be made available on request to the ALSPAC Executive via this website, which also provides full details and distributions of the ALSPAC study variables: http://www.bristol.ac.uk/alspac/researchers/access/. The ALSPAC data management plan (available here: http://www.bristol.ac.uk/alspac/researchers/data-access/documents/alspac-data-management-plan.pdf) describes in detail the policy regarding data sharing. A sampler set of similar data containing relevant ALSPAC variables is available from the European Genome-phenome Archive (accession number: EGAS00001000090): https://www.ebi.ac.uk/ega/studies/EGAS00001000090.

## Supplementary material

Abbreviations used in supplementary files: Vq, the amount of variance the genetic instrument explains of the maternal exposure; Vo, the amount of variance the same genetic variants (as those used in the maternal IV) in the offspring explains of the offspring outcome; Vf, the amount of variance the same genetic variants (as those used in the maternal IV) in the father explains of the offspring outcome; beta XY, the true causal effect of exposure X (in mothers) on outcome Y (in offspring); beta IV (median), the median of the simulated IV estimates of the effect of exposure X (in mothers) on outcome Y (in offspring); SE (beta IV), the standard error of the Beta IV (IV estimate of causal effect).

**Supplementary File 1: Additional methods.** This file includes additional methodological information for the following:

*Section 1.* The rationale for our assertion that in some very specific situations violation of the exclusion restriction criteria via offspring genotype can be corrected by subtracting 0.5 from the unadjusted IV estimate. We provide the specific criteria required for this to be valid and show the Directed Acyclic Graph underlying this correction.

*Section 2.* Details of simulation studies for sensitivity analyses of the effect of being able (or not) to adjust for offspring and/or paternal genotype.

*Section 3.* R-code for these simulation studies.

*Section 4.* Details of the methods used in the illustrative example from the Avon Longitudinal Study of Parents and Children (ALSPAC).

*Table S1.* Association of 97-SNP weighted allele score IV for maternal pre-pregnancy BMI with maternal, paternal and offspring confounders. Relevant to the illustrative ALSPAC example.

**Supplementary File 2: Full simulation results for strong IV with a true negative effect.** This provides all results of the simulation studies in the example with the IV is strong and the true causal effect is negative (inverse).

**Supplementary File 3: Full simulation results for strong IV with a true null effect.** This provides all results of the simulation studies in the example with the IV is strong and the true causal effect is null.

**Supplementary File 4: Full simulation results for strong IV with a true positive effect.** This provides all results of the simulation studies in the example with the IV is strong and the true causal effect is positive.

**Supplementary File 5: Full simulation results for weak IV with a true negative effect.** This provides all results of the simulation studies in the example with the IV is weak and the true causal effect is negative (inverse).

**Supplementary File 6: Full simulation results for weak IV with a true null effect.** This provides all results of the simulation studies in the example with the IV is weak and the true causal effect is null.

**Supplementary File 7: Full simulation results for weak IV with a true positive effect.** This provides all results of the simulation studies in the example with the IV is weak and the true causal effect is positive.
