# Supplementary material for: Using Mendelian randomization to determine causal effects of maternal pregnancy (intrauterine) exposures on offspring outcomes: Sources of bias and methods for assessing them
Source: Wellcome Open Res. 2017 Feb 14;2:11. [Version 1] doi: 10.12688/wellcomeopenres.10567.1 (PMC5386135; doi:10.12688/wellcomeopenres.10567.1)
Supplement: Supplementary file 1 [file wellcomeopenres-2-11387-s0000.tgz › b0e8041c-a20b-47ed-a336-6964eb5b0bf7.docx]

**Supplementary File 1:**

**Using Mendelian randomization to determine causal effects of maternal pregnancy exposures on offspring outcomes: sources of bias and methods for assessing them.**

Debbie A Lawlor, Rebecca Richmond, Nicole Warrington, George McMahon, George Davey Smith, Jack Bowden, David M Evans.

| **Section** | **Description** | **Pages** |
| --- | --- | --- |
| 1 | Correction of the unadjusted (for offspring and paternal genotype) MR estimate where there is concern that the exclusion restriction criteria may be violated by offspring genotype. | 2-7 |
| 2 | Details of simulation studies for sensitivity analyses of the effect of being able (or not) to adjust for offspring and/or paternal genotype | 8-10 |
| 3 | R code for simulation studies | 11-14 |
| 4 | Details of the methods used in the illustrative example from the Avon Longitudinal Study of Parents and Children | 15-18 |
| Table S1 | Association of 97-SNP weighted allele score IV for maternal pre-pregnancy BMI with maternal, paternal and offspring confounders. | 19 |
|  | References | 20 |

**1. Correction of the unadjusted (for offspring and paternal genotype) MR estimate where there is concern that the exclusion restriction criteria may be violated by offspring genotype.**

In a very specific situation where, amongst other criteria the maternal exposure and offspring outcomes are **exactly** the same (e.g. testing the causal effect of maternal pregnancy BMI on offspring BMI) a simple correction of the unadjusted IV analysis of maternal genetic variants on offspring outcome that involves subtracting 0.5 may yield an asymptotically unbiased estimate of the causal effect of maternal exposure on child’s outcome. **Figure S1** shows the rationale for this.

**Figure S1: Rational for the simple solution to violation of the exclusion restriction criteria via offspring genotype in a very specific situation**


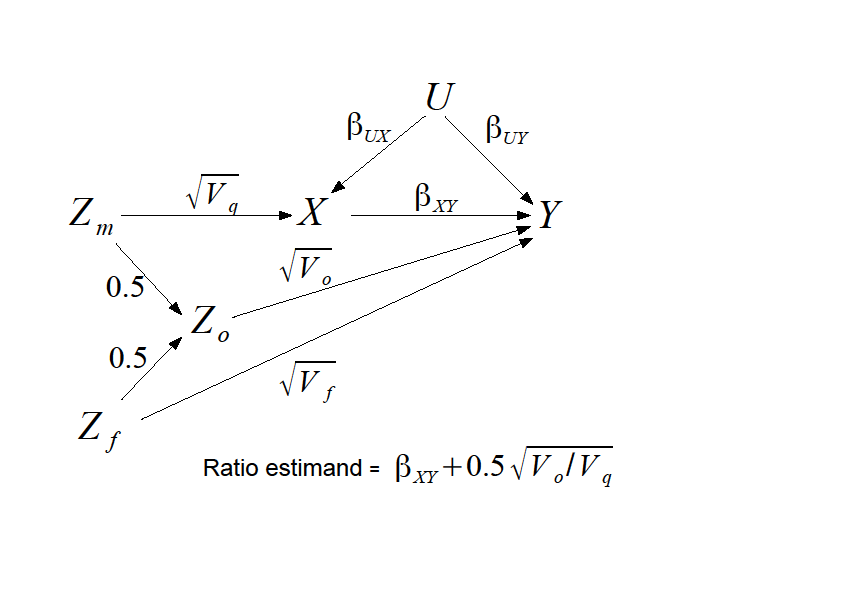


Where:

*X* is the exposure that is measured in the mother and hypothesised to affect the outcome *Y* in the offspring. We are interested in testing this causal effect using an instrumental variable analysis with a maternal genetic variant (*Z_m_*). If X and Y are the same measure (e.g. *X* = maternal pregnancy BMI and *Y* = offspring BMI assessed in adulthood), then we know that the exclusion restriction assumption will be violated via offspring (and paternal) genotype as shown in the figure.

*Z_m_* is the maternal genetic instrumental variable (i.e. a genetic variant that is robustly associated with *X*) and *Zo* the equivalent genetic variant in the offspring and *Z_f_* the equivalent genetic variant in the father.

*U* represents (unmeasured) confounders of the association of maternal exposure *X* with offspring outcome *Y*, with *β_UX_* and *β_UY_* representing their associations with *X* and *Y*, respectively.

$\sqrt{V_{q}}$ quantifies the association between the maternal genetic variant and her exposure *X* (i.e. for each unit increase in the maternal genetic variant, we expect that the exposure *X* would change by $\sqrt{V_{q}}$ units. The square of this path coefficient (i.e. *V_q_*) represents the amount of variance the genetic instrument explains in the maternal exposure and therefore quantifies the instrumental variable strength, $\sqrt{V_{o}}$ quantifies the association between the offspring genetic variant and their outcome *Y* and $\sqrt{V_{f}}$quantifies the association between the father’s genetic variant and the offspring outcome *Y*

*β_XY_* represents the causal effect of maternal exposure X on Y that we estimate from the ratio instrumental variable MR analyses.

However we know (as shown in the figure) that this will be biased because of the path from the maternal genetic instrument *Z_m_* via the same genetic variant in offspring *Z_o_*, which influences offspring outcome Y. If we do not adjust for *Z_o_*, and assuming the absence of assortative mating, the path from father’s genetic variant *Z_f_* to maternal instrumental variable *Z_m_* is blocked at the collider *Z_o_*. We know that the expected correlation of a bi-allelic genetic variant in either mother or father with the same genetic variant in their offspring is 0.5, and this is shown in the figure. Thus the magnitude of the bias via the offspring genetic variant is 0.5 multiplied by $\frac{\sqrt{V_{O}}}{\sqrt{V_{q}}}$as shown in the figure. If $\sqrt{Vo}$ and $\sqrt{V_{q}}$ are the same – as should be the case asymptotically when *X* and *Y* are the same trait, then the bias is exactly 0.5.

The criteria that are required for this simple correction are, however, only likely to be met in rare circumstances:

1. The maternal exposure and offspring outcome are exactly the same and are a continuously measured variable. The criteria that the offspring outcome and maternal exposure are exactly the same may also mean that these need to be assessed at a similar age (see example described in relation to Figure S2 below).
2. The association of mother’s genetic instrument with her exposure and of the same genetic variant in offspring with their outcome are identical. That is they are scaled the same way and have the same underlying regression coefficient. This requirement means that genetic variants used as maternal instruments have the same magnitudes of effect on her exposure in pregnancy as they do outside of pregnancy (as most offspring will not be pregnant and they will be approximately 50% male). As outcomes in offspring may be measured in infancy, childhood, adolescence or adulthood whereas pregnant women will be mostly adults, it also requires that the magnitudes of association of the genetic variants with maternal exposure/offspring outcome do not vary by age. For example, there is some evidence that genetic variants for BMI vary in their magnitude of association with BMI depending on whether it is measured in infancy, childhood or adulthood.(1, 2)
3. There must be no other violations of the exclusion restriction criteria (e.g. no paths from mother’s genetic instrument via pre-conceptual or post-natal mechanisms or horizontal pleiotropy). Where multiple genetic instruments are used (as is often the case in MR) this applies for each of these individually.
4. Other assumptions of MR (robust association of maternal genetic instrument with her exposure and no relationship of confounders of the *X*-*Y* association with the maternal genetic instrument) must not be violated. Where multiple genetic instruments are used (as is often the case in MR) this applies for each of these individually.
5. This approach assumes linearity and additivity and therefore is not suitable for ratio estimates (e.g. odds ratios, risk ratios, ratios of geometric means).
6. Where multiple genetic instruments are used (as is often the case in MR) this approach assumes there is the same underlying regression (same magnitude and direction) between each maternal variant and maternal pregnancy exposure as between offspring variant and offspring outcome. If there are doubts about this, for example, if it were plausible that some of the maternal genetic instruments had effects that differed with age, and mothers and offspring had different age distributions, it may be possible to correct the ratio separately for each variant (using 0.5 × $\sqrt{Vo}/Vq$) and then meta-analyse each separate result.

This approach, and the criteria we list above, apply to large samples. Currently, we do not know what the effect of sampling variation, nor of measurement error, would have on the validity of this simple approach. Therefore, we would caution against its use as a main analysis and use it only when the above criteria are likely to be in place with sensitivity analyses.

The intuition behind this simple adjustment for this special case can be explained in reference to **Figure S2** which shows an example of a plausible research question (does exposure to different intrauterine levels of maternal gestational BMI effect postnatal offspring BMI?) . In this example the association between maternal genetic IV and offspring outcome (BMI) is expected to be $\sqrt{V_{q}}\times\beta_{XY} +0.5\times\sqrt{V_{O}}$ (i.e. the sum of the direct path through the mother’s intrauterine BMI and the indirect path through the offspring’s genotype and its relation to their BMI; this would be obtained asymptotically if we regressed offspring outcome on maternal genotype with no adjustments). The expected association between maternal genetic IVs and maternal BMI is $\sqrt{V_{q}}$ (which would be obtained asymptotically if we regressed maternal BMI on maternal genetic IVs). Therefore asymptotically the Wald estimate obtained by taking the ratio of these two associations is expected to be $\frac{\sqrt{V_{q}}\times\beta_{XY} +0.5\times\sqrt{V_{O}}}{\sqrt{V_{q}}}=\beta_{XY} +0.5 \times\frac{\sqrt{V_{O}}}{\sqrt{V_{q}}}$. In the special situation described above where the association between mother’s genetic instrument with her exposure and the association between the same genetic variant in offspring with their outcome are identical (i.e. $\sqrt{V_{q}}=\sqrt{V_{O}}$), as we might expect in the example showing in **Figure S2**, particularly if offspring BMI is assessed in their adulthood, this expression simplifies to $\beta_{XY} +0.5$. It follows that subtracting 0.5 from this estimate will give a valid estimate of $\beta_{XY}$, the true causal effect of maternal exposure (BMI) on offspring outcome (BMI), if the criteria listed above are met.

**Figure S2: Directed Acyclic Graph of the intrauterine effect of exposure to different levels of maternal gestational BMI on offspring adult BMI**

A key criteria for this approach to work is that maternal exposure and offspring outcome are exactly the same, such that the (maternal) genetic IV association with maternal exposure is exactly the same as the equivalent association of the offspring’s same allele score with the offspring outcome of interest. If offspring BMI is assessed in adulthood then this association is likely to be the case, as the BMI variants will relate to BMI with the same magnitude in adult mothers and their offspring when BMI is assessed in adulthood. There is evidence that some genetic variants have age specific effects, such that their magnitude of association with BMI varies between infancy, childhood and adulthood.(1, 2) Thus, if offspring BMI is measured in infancy or early childhood the magnitude of a genetic BMI allele score with BMI may differ between pregnant adult women and their infant/childhood offspring. Nonetheless we would still anticipate some positive correlation between the two.

**2. Details of simulation studies for sensitivity analyses of the effect of being able (or not) to adjust for offspring and/or paternal genotype**

The rationale for these simulation sensitivity analyses together with a discussion of their results are presented in Section 3.4 of the main paper. Here we provide details of the methods including R code.

For each simulation scenario, we generated 1000 replicates consisting of 10000 parent offspring trios. For each replicate we generated maternal, paternal and offspring genotypes at a single genetic locus (even if not all of these were used in the analysis) assuming a trait increasing allele frequency of q = 0.8 and standard autosomal Mendelian inheritance. From these genotypes, latent values for maternal (Z_m_), paternal (Z_f_) and offspring (Z_o_) genotypes were obtained assuming additivity and unit variance. The maternal exposure variable (X) for each family *i*, was generated using the following equation:

$$X_{i}=\sqrt{V_{q}}\times{Z_{m}}_{i}+\beta_{UX}\times U_{i}+\delta_{i}$$

where V_q_ denotes the variance in the maternal exposure explained by mother’s genotype, Z_m_ is a latent variable of unit variance indexing the maternal genotype, U is a standard normal random variable representing all confounding influences, β_UX_ denotes the total effect of latent confounders U on the maternal exposure X, and δ is a random normal variate with mean zero and variance needed to ensure that X has unit variance asymptotically.

The offspring phenotype (Y) for each family *i* was generated according to the following model:

$$Y_{i}=\beta_{XY}\times X_{i}+\beta_{UY}\times U_{i}+\sqrt{V_{o}}\times{Z_{o}}_{i}+\sqrt{V_{f}}\times{Z_{f}}_{i}+\varepsilon_{i}$$

where β_XY_ is the causal effect of the maternal exposure X on offspring outcome Y, β_UY_ is the total effect of confounding variables on offspring outcome, V_o_ denotes the variance in the offspring outcome explained by offspring genotype, Z_o_ is a latent variable of unit variance indexing the offspring genetic instrument, V_f_ denotes the variance in the offspring outcome explained by paternal genotype, Z_f_ is a latent variable of unit variance indexing the paternal genetic instrument, and ε is a random normal variate with mean zero and variance needed to ensure that Y has unit variance asymptotically. The underlying data generating model is represented by the following DAG (**Figure S3**)

**Figure S3: Data generating model for simulation sensitivity analyses**


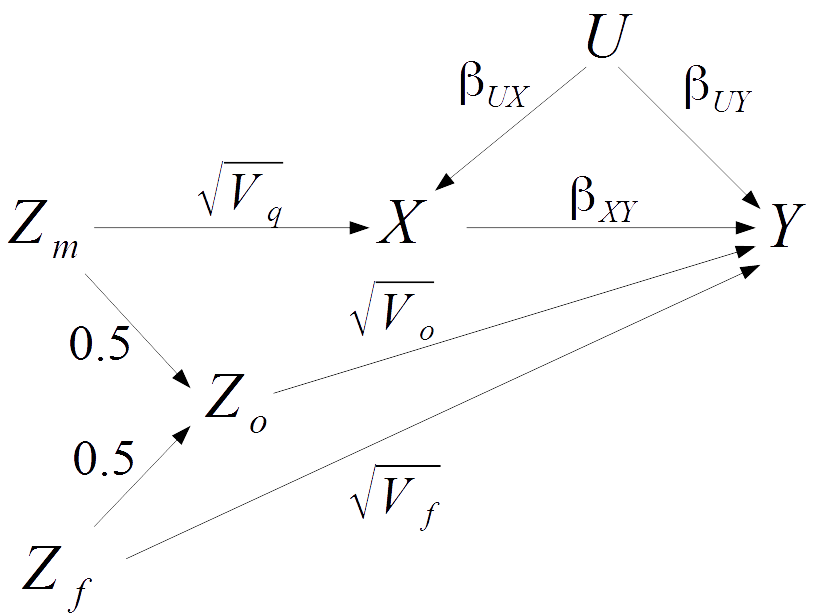


We examined the effect of (1) strong or weak instruments for the maternal exposure (two conditions: V_m_ = 2% or V_m_ = 0.05%), (2) the strength of the causal relationship between the maternal exposure and offspring outcome (three conditions: β_XY_ = -0.1, β_XY_ = 0, or β_XY_ = 0.1), (3) the strength of confounding between the maternal exposure and offspring outcome (three conditions: β_UX_ = β_UY_ = -0.5, β_UX_ = β_UY_ = 0, β_UX_ = β_UY_ = 0.5), (4) offspring genotype on offspring outcome (five conditions: V_o_ = 0, V_o_ = 0.005, V_o_ = 0.01, V_o_ = 0.02, V_o_ = 0.05), (5) paternal genotype on offspring outcome (five conditions: V_f_ = 0, V_f_ = 0.005, V_f_ = 0.01, V_f_ = V_f_ = 0.02, V_f_ = 0.05), and all combinations of these factors. We recorded the median instrumental variables estimate of the causal relationship, it's standard error and the power/type 1 error rate of the analysis.

**3. R code used for the simulation studies**

#Simulate Instrumental Variable (Zm), Mother's exposure (Xm), Offspring outcome (Ym)

#Also a path to Ym through offspring genotype

#Also a path from father's genotype to offspring outcome

library(sem) #Install two-stage least squares library

simulate_IV <- function(Nrep = 1000, N = 10000, Vq = 0.01, p = 0.2, BetaXY = 0.1, BetaUX = 0, BetaUY = 0,

Vo = 0, Vf = 0) {

#Nrep = Number of simulation replicates

#N = Sample size

#Vq = Variance explained by instrument on intermediate

#p = Decreaser allele frequency

#BetaXY = Causal effect of X on Y

#BetaUX = Confounding effect of U on X

#BetaUY = Confounding effect of U on Y

#Vo = Variance explained by offspring genotype on outcome

#Vf = Variance explained by father's genotype on offspring outcome

q <- 1-p #Increaser allele frequency

beta1 <- vector(length = Nrep)

beta2 <- vector(length = Nrep)

beta3 <- vector(length = Nrep)

beta4 <- vector(length = Nrep)

pval1 <- vector(length = Nrep)

pval2 <- vector(length = Nrep)

pval3 <- vector(length = Nrep)

pval4 <- vector(length = Nrep)

a <- sqrt(1/(2*p*q)) #Create genetic variable of variance one

Ve <- (1- Vq - BetaUX^2) #Residual variance in intermediate

sde <- sqrt(Ve)

for(j in 1:Nrep) {

#Sample mothers' genotypes

Zm <- sample(x = c(-a,0,a), size = N, replace = TRUE, prob = c(p^2, 2*p*q, q^2))

#Sample fathers' genotypes

Zf <- sample(x = c(-a,0,a), size = N, replace = TRUE, prob = c(p^2, 2*p*q, q^2))

#Simulate offspring genotype

Zo <- vector(length = N)

#Mother's untransmitted haplotype

Zu <- vector(length = N)

r <- runif(N)

for (i in 1:N) {

if((Zm[i]==-a) && (Zf[i]==-a)) {Zo[i] = -a; Zu[i] = 0}

if((Zm[i]==-a) && (Zf[i]==0)) {if(r[i] <= 0.5) {Zo[i] = -a; Zu[i] = 0}

else {Zo[i] = 0; Zu[i] = 0}}

if((Zm[i]==-a) && (Zf[i]==a)) {Zo[i] = 0; Zu[i] = 0}

if((Zm[i]==0) && (Zf[i]==-a)) {if(r[i] <= 0.5) {Zo[i] = -a; Zu[i] = 1}

else {Zo[i] = 0; Zu[i] = 0}}

if((Zm[i]==0) && (Zf[i]==0)) {

if(r[i] <= 0.25) {Zo[i] = -a; Zu[i] = 1

if(r[i] > 0.25 && r[i] <= 0.50) {Zo[i] = 0; Zu[i] = 1}

if(r[i] > 0.50 && r[i] <= 0.75) {Zo[i] = 0; Zu[i] = 0}

if(r[i] > 0.75) {Zo[i] = a; Zu[i] = 0}

}

if((Zm[i]==0) && (Zf[i]==a)) {if(r[i] <= 0.5) {Zo[i] = a; Zu[i] = 0}

else {Zo[i] = 0; Zu[i] = 1}}

if((Zm[i]==a) && (Zf[i]==-a)) {Zo[i] = 0; Zu[i] = 1}

if((Zm[i]==a) && (Zf[i]==0)) {if(r[i] <= 0.5) {Zo[i] = a; Zu[i] = 1}

else {Zo[i] = 0; Zu[i] = 1}}

if((Zm[i]==a) && (Zf[i]==a)) {Zo[i] = a; Zu[i] = 1}

}

}

#Simulate mothers' intermediates

U <- rnorm(N, mean = 0, sd = 1)

Xm <- sqrt(Vq)*Zm + BetaUX*U + rnorm(N, mean = 0, sd = sde)

#Simulate offspring outcome

residvar = 1 - (BetaXY^2 + 2*BetaXY*BetaUX*BetaUY + BetaUY^2 + Vo + sqrt(Vo)*sqrt(Vq)*BetaXY + Vf +

sqrt(Vo)*sqrt(Vf)) #Residual variance for outcome so adds up to one

Ym <- BetaXY*Xm + BetaUY*U + sqrt(Vo)*Zo + sqrt(Vf)*Zf + rnorm(N,0,sqrt(residvar))

temp <- cbind(Ym, Xm, Zm, Zu, Zo, Zf)

#Need to create this intermediate variable for tsls function to recognise the data

#Perform 2SLS regression without correction for offspring genotype

results1 <- summary(tsls(Ym ~ Xm, ~ Zm, data = temp))

#Perform 2SLS regression with correction for offspring genotype

results2 <- summary(tsls(Ym ~ Xm + Zo, ~ Zm + Zo, data = temp))

#Perform 2SLS regression using untransmitted maternal allele

results3 <- summary(tsls(Ym ~ Xm, ~ Zu, data = temp))

#Perform 2SLS regression with correction for offspring genotype AND father's genotype

results4 <- summary(tsls(Ym ~ Xm + Zo + Zf, ~ Zm + Zo + Zf, data = temp))

#Gather results

beta1[j] <- results1$coefficients[2,1]

pval1[j] <- results1$coefficients[2,4]

beta2[j] <- results2$coefficients[2,1]

pval2[j] <- results2$coefficients[2,4]

beta3[j] <- results3$coefficients[2,1]

pval3[j] <- results3$coefficients[2,4]

beta4[j] <- results4$coefficients[2,1]

pval4[j] <- results4$coefficients[2,4]

}

results <- cbind(beta1, beta2, beta3, beta4, pval1, pval2, pval3, pval4)

print(results)

write.table(results, paste("SimResults-Vq", Vq, "-p", p, "-BetaXY", BetaXY, "-BetaUX", BetaUX,

"-BetaUY", BetaUY, "-Vo", Vo, "-Vf", Vf, ".txt", sep=""), quote=F, row.names=F)

}

**4. Details of the methods used in the illustrative example from the Avon Longitudinal Study of Parents and Children**

Data from the Avon Longitudinal Study of Parents and Children (ALSPAC) are used as an illustrative example of how to work through the recommendations that we list in the main paper for using MR to test causal intrauterine effects of maternal pregnancy exposures on offspring postnatal outcomes. Here we provide full details of the methods used to produce the results for that illustrative example. Results are presented and discussed in the main paper.

***3.1 Study population***

ALSPAC is a prospective population-based birth cohort study that recruited 14,541 pregnant women resident in Avon, UK with expected dates of delivery from 1^st^ April 1991 to 31^st^ December 1992 (<http://www.alspac.bris.ac.uk>.).(3, 4) Ethical approval was obtained from the ALSPAC Law and Ethics committee and relevant local ethics committees and all women provided informed written consent. Please note that the study website contains details of all the data that is available through a fully searchable data dictionary:

http://www.bris.ac.uk/alspac/researchers/data-access/data-dictionary/

Full details of how maternal BMI, offspring BMI and FMI and genetic data were obtained have been published previously.(3-6) **Figure S4** shows the directed acyclic graph that informed our analyses. Multivariable regression, with adjustment for potential confounders was undertaken to explore the association of maternal BMI with offspring BMI and FMI at age 18 years, and to enable us to compare the different MR approaches that we used to explore the causal effect of maternal pregnancy BMI on offspring BMI and FMI at age 18.

**Figure S4: Directed acyclic graph of associations between characteristics in the real data (ALSPAC) example**

***3.2 Main MR analyses***

We used two complementary approaches for our main MR analyses. First, we used a weighted allele score of 97 genetic variants that have been found to be robustly associated with BMI,(7) along with adjustment for the same weighted allele score in offspring. The weights that were used are largely external; they were taken from the regression coefficients of the latest GWAS consortia (ALSPAC was included in that consortia but contributed less than 2% of the 322,154 participants included in that study).(7) The weighted allele score was derived as:

$$Weighted BMI score=w_{1}\times\mathrm{SNP}_{1}+w_{2}\times\mathrm{SNP}_{2}+\ldots w_{n}\times\mathrm{SNP}_{n}$$

Where w is the weight (i.e. the beta-coefficient of association of the SNP with BMI from the published GWAS) and SNP is the dosage of BMI-raising alleles at that locus (i.e. 0, 1 or 2 BMI raising alleles). The score was then rescaled to reflect the average number of BMI-increasing alleles carried by an individual using the formula described in Lin *et al* (8):

$$Rescaled weighted BMI score=\frac{Weighted score \times Number of SNPs available}{Sum of weights of available SNPs}$$

We used TSLS instrumental variable analysis,(9) with this weighted allele score as the instrument, for this MR approach. For illustrative purposes we also present the unadjusted (for offspring genotype) results from this method.

Second, we examined the causal effect using the non-transmitted (to offspring) haplotype approach, as described by Zhang and colleagues.(10) To implement this method, we inferred maternal-offspring allelic transmissions based on either direct comparison of genotypes (between mothers and their offspring), or where transmissions could not be determined unequivocally at a single locus, with the assistance of surrounding haplotypes phased in the ALSPAC mothers and children. We constructed allelic scores based on either transmitted or non-transmitted alleles and performed IV regression analyses of these allele scores on offspring BMI.

***3.3 Sensitivity analyses with real data***

We used MR-Egger and weighted median methods applied to the maternal weighted allele score with adjustment for offspring allele score in sensitivity analyses. As these methods are not directly comparable to the first main IV analysis which used TSLS (a common method used in one-sample MR)(9), we also present the MR results from the IVW MR approach. In all three of these approaches the IVs were 97 maternal BMI increasing variants adjusted for the same 97 variants in offspring (with both externally weighted using the published GWAS coefficients as weights(7) to which ALSPAC contributed a small proportion of data, as described above).

Since this example may fulfil the criteria for the ‘special’ case that we describe above in Section 1, where maternal exposure and offspring outcome are the same, we also undertook an additional sensitivity analysis which was to deduct 0.5 from the unadjusted MR estimate and each value of its 95% confidence interval.

***3.4 Sensitivity analyses using simulated data***

We undertook simulation studies using the methods described above in Section 2. In these analyses we examined results assuming a true null result and also a true positive effect of 0.2SD change in the offspring BMI/FMI per 1SD change in maternal pregnancy BMI for the MR approaches equivalent to our two main analyses (maternal weighted allele score adjusted for offspring identical weighted allele score and using the maternal non-transmitted allele) and in addition the maternal weighted allele score adjusted for both offspring and paternal identical weighted allele score. We additionally fixed all confounders of the offspring outcome to zero, the magnitude of association of maternal genetic instruments with her BMI (i.e. instrument strength) and offspring genotypes with their BMI to R=0.02 (7), and the magnitude of paternal genotypes with offspring BMI to R= 0.01.

**Table S1: Association of 97-SNP weighted allele score IV for maternal pre-pregnancy BMI with maternal, paternal and offspring confounders.**

| **Confounder (Unit of categories)^a^** | **N^b^** | **Difference in mean weighted maternal allele score (SD) per confounder unit or category increase (95%CI)** | **N^b^** | **Difference in mean weighted maternal allele score (SD) per confounder unit or category increase, with adjustment for offspring allele score (95%CI)** |
| --- | --- | --- | --- | --- |
| Household social class (I, II, IIINM, IIIM, IV, V) | 4328 | 0.038 (0.008, 0.067) | 4328 | 0.027 (0.001, 0.052) |
| Maternal education (CSE/vocational, O, A, University degree) | 4353 | -0.018 (-0.048, 0.012) | 4353 | 0.001 (-0.026, 0.028) |
| Paternal education (CSE/vocational, O, A, University degree) | 4141 | -0.027 (-0.054, 0.001) | 4141 | -0.025 (-0.050, 0.001) |
| Parity (0, 1, 2, 3+) | 4523 | 0.032 (0.000, 0.065) | 4523 | 0.027 (-0.002, 0.055) |
| Maternal smoking (never, early pregnancy only, through pregnancy) | 4391 | 0.019 (-0.020, 0.057) | 4391 | 0.009 (-0.025, 0.044) |
| Offspring smoking at 18 (no, current) | 1220 | 0.058 (-0.05, 0.17) | 1220 | -0.020 (-0.13, 0.86) |
| Paternal BMI (kg/m^2^) | 3387 | 0.002 (-0.009, 0.012) | 3387 | -0.014 (-0.024, -0.005) |

^a^ For categorical confounders results are per category increase; Household social class categories: I: Professionals; II Managerial and technical; IIINM skilled non-manual; IIIM skilled manual; IV semi-skilled; V unskilled (the category represents the highest level occupation of mother or her partner, whichever his highest); Education categories: CSE certificate of secondary education normally taken at age 15/16 lowest level, O ordinary level normally taken at 15/16 higher level that CSE, A advanced level normally taken at age 18, university degree.

^b^ For these analyses maximal numbers of participants for each confounder who have both maternal and offspring allele score are included (numbers vary by confounder).

CI: confidence interval.

**References**

1. Warrington NM, Howe LD, Paternoster L, Kaakinen M, Herrala S, Huikari V, et al. A genome-wide association study of body mass index across early life and childhood. Int J Epidemiol. 2015;44(2):700-12.

2. Felix JF, Bradfield JP, Monnereau C, van der Valk RJ, Stergiakouli E, Chesi A, et al. Genome-wide association analysis identifies three new susceptibility loci for childhood body mass index. Hum Mol Genet. 2016;25(2):389-403.

3. Boyd A, Golding J, Macleod J, Lawlor DA, Fraser A, Henderson J, et al. Cohort Profile: The 'Children of the 90s'--the index offspring of the Avon Longitudinal Study of Parents and Children. Int J Epidemiol. 2013;42(1):111-27.

4. Fraser A, Macdonald-Wallis C, Tilling K, Boyd A, Golding J, Davey Smith G, et al. Cohort Profile: The Avon Longitudinal Study of Parents and Children: ALSPAC mothers cohort. Int J Epidemiol. 2013;42(1):97-110.

5. Lawlor DA, Timpson N, Harbord RM, Leary S, Ness A, McCarthy MI, et al. Exploring the developmental overnutrition hypothesis using parental-offspring associations and the *FTO* gene as an instrumental variable for maternal adiposity. The Avon Longitudinal Study of Parents and Children (ALSPAC). PloS Medicine. 2008;5:e33.

6. Richmond RC, Timpson NJ, Felix JF, Palmer T, Gaillard R, McMahon G, et al. Using Genetic Variation to Explore the Causal Effect of Maternal Pregnancy Adiposity on Future Offspring Adiposity: A Mendelian Randomisation Study. PLoS Med. 2017;14(1):e1002221.

7. Locke AE, Kahali B, Berndt SI, Justice AE, Pers TH, Day FR, et al. Genetic studies of body mass index yield new insights for obesity biology. Nature. 2015;518(7538):197-206.

8. Lin X, Song K, Lim N, Yuan X, Johnson T, Abderrahmani A, et al. Risk prediction of prevalent diabetes in a Swiss population using a weighted genetic score-the CoLaus Study. Diabetologia. 2009;52(4):600-8.

9. Palmer TM, Sterne JA, Harbord RM, Lawlor DA, Sheehan NA, Meng S, et al. Instrumental variable estimation of causal risk ratios and causal odds ratios in Mendelian randomization analyses. Am J Epidemiol. 2011;173(12):1392-403.

10. Zhang G, Bacelis J, Lengyel C, Teramo K, Hallman M, Helgeland O, et al. Assessing the Causal Relationship of Maternal Height on Birth Size and Gestational Age at Birth: A Mendelian Randomization Analysis. PLoS Med. 2015;12(8):e1001865.
