# Supplementary material for: Using Mendelian randomization to determine causal effects of maternal pregnancy (intrauterine) exposures on offspring outcomes: Sources of bias and methods for assessing them
Source: Wellcome Open Res. 2017 Feb 14;2:11. [Version 1] doi: 10.12688/wellcomeopenres.10567.1 (PMC5386135; doi:10.12688/wellcomeopenres.10567.1)
Supplement: Peer review history

# Peer review: Referee response for version 1

**DOI:** 10.21956/wellcomeopenres.11387.r20454

**Published:** 2017-03-22

**Authors:** Sridharan Raghavan

**Affiliations:** 1 Department of Veterans Affairs, Eastern Colorado Healthcare System, Denver, CO, USA

The article addresses the important methodological issues surrounding application of genetic instrumental variable (IV) analysis, or Mendelian randomization, to maternal-child exposure-outcome associations. The authors describe scenarios in which the so-called exclusion restriction might be violated and then describe analytic approaches to address the potential bias in IV estimates that could arise. The article is clearly written with apt examples and is very thorough, especially with the abundant supplemental materials including code. This manuscript is likely to be valuable to the community of researchers interested in IV applications to maternal-child associations.

 There are several issues that it would be helpful if the authors could address to make the manuscript a more comprehensive resource.

  

1. The authors describe the usefulness of examining associations between the maternal genetic IV and measured confounders under several different conditions (page 4). However, it is not clear how to optimally address associations between the genetic IV and measured confounders (see comment 2a below) as the bias introduced by such IV-confounder associations on the IV-outcome association estimate are not always clear. ^1^
2. It would help if the authors could specifically address adaptation of their simulation-based sensitivity analyses to examine pleiotropy. Two examples that came to mind when reading the manuscript:
   
  1. In the example evaluating a genetic IV to examine the association between maternal pregnancy BMI and childhood BMI, the authors describe associations between the IV and measured confounders (social class and parity). Depending on how the DAG is conceived, one could see this as an example of pleiotropy leading to a violation of the IV assumption that all directed paths from the IV to the outcome pass through maternal pregnancy BMI (especially for parity if there is a causal association between BMI genetic variants and parity). Furthermore, there might be plausible paths connecting the BMI IV, maternal pregnancy BMI, parity, and the outcome that would suggest that bias could be introduced by including parity as a covariate in either the multivariable regression or the IV analysis. Could the authors address their choice to include social class but not parity in their multivariable regression model and discuss adaptations of their simulations to address possible contributions of pleiotropy to IV estimates?
  2. The authors’ discussion of assortative mating is clear. However, they do not address the scenario in which the assortment occurs not on the exposure of interest but on a correlated trait (e.g. if the exposure of interest is maternal diabetes, IV is a genetic risk score for diabetes, outcome of interest is childhood BMI or glucose levels, and assortative mating might occur on the basis of maternal/paternal BMI). In this scenario, accounting for paternal and offspring diabetes genetic variants is unlikely to block all paths between the IV and outcome that do not go through the exposure of interest, and adding covariates related to assortment (maternal and/or paternal BMI) might induce bias in the IV-outcome association estimate. While this is essentially a special case of pleiotropy, it may be worth making the distinction between assortment based on the exposure of interest (for which the IV is selected) and assortment based on traits correlated with the exposure of interest. The impact of the latter case on the IV-outcome estimate can likely be addressed through simulation as well.
3. The authors do not include offspring genetic variants for BMI in their multivariable regression model in the example derived from ALSPAC data. This would seem to inflate the difference in estimates of exposure-outcome association compared to the IV-outcome association (since offspring genetic variants are included in the IV analysis but not in the multivariable regression). While this does not affect the causal effect estimate from the IV analysis, it does impact any statistical test for difference in effect estimates comparing the multivariable regression model and the IV model. Can the authors address this discrepancy?
4. Two additional sources of bias in IV estimates that are not unique to maternal-offspring IV analyses arise from additional assumptions ^2^ that the authors do not describe in their manuscript: 1. The assumption of no effect modification by the instrument on the exposure-outcome association; 2. The assumption of monotonicity between instrument and exposure. In the context of genetic instruments for which we infrequently understand the causal variants or biological pathways involved, the assumption of no effect modification is difficult to verify. Similarly, when a genetic instrument is used for a dichotomous exposure (e.g. diabetes or obesity), the monotonicity assumption is not always satisfied (some mothers with a heavy burden of diabetes-associated genetic variants might not develop pre-pregnancy or gestational diabetes). It might be helpful to include a brief discussion of sources of bias common to all genetic IV analyses, irrespective of whether an intrauterine exposure is of interest, that warrant consideration.

Minor comments:

1. Typo- Para 1: sentence beginning “For example, IADSPG…. ***through ***antenatal care.”
2. Intro, last para, sentence that starts “However, the majority ….”, there is an incorrect comma.
3. Extraneous comma in the last sentence of para starting “ **True pleiotropic effects of the maternal genetic instrumental variable**.”

I have read this submission. I believe that I have an appropriate level of expertise to confirm that it is of an acceptable scientific standard.

## References

[1] JacksonJWSwansonSA: Toward a clearer portrayal of confounding bias in instrumental variable applications. *Epidemiology*.2015;26(4) : 10.1097/EDE.0000000000000287 498-504 10.1097/EDE.0000000000000287 25978796PMC4673662

[2] HernánMARobinsJM: Instruments for causal inference: an epidemiologist's dream?. *Epidemiology*.2006;17(4) : 10.1097/01.ede.0000222409.00878.37 360-72 10.1097/01.ede.0000222409.00878.37 16755261

# Peer review: Referee response for version 1

**DOI:** 10.21956/wellcomeopenres.11387.r20276

**Published:** 2017-03-21

**Authors:** Lorenzo Richiardi, Maja Popovic

**Affiliations:** 1 Cancer Epidemiology Unit-CeRMS, Department of Medical Sciences, University of Turin, Turin, Italy; 2 Department of Medical Sciences, University of Turin, Turin, Italy

This is a clear and balanced article on the use of MR to analyse the effects of maternal pregnancy exposures on offspring outcomes. Our comments are reported below.

**1) Introduction**

 a. ”However, evidence that the proxy measures used by IADPSG developers to indicate offspring obesity (high birth weight, birth skinfolds and cord-blood c-peptide) are accurate predictors of future risk of childhood obesity, or that treating women with the IADPSG criteria will effectively reduce childhood obesity, is lacking”. It would be important to clearly distinguish between prediction and the possibility to prevent obesity through interventions, as the former concept does not require causation.

 b. “In other areas women’s lifestyle in pregnancy is potentially being blamed for all future health risks in her offspring”. This statement is maybe too strong; in addition strong evidence of causation (or lack of causation) would not solve the blaming component. 

 c. “However, conventional methods are unlikely to be suitable [to provide valid causal statements]”. Also this statement is maybe too strong. We agree that residual confounding is a major issue for causal inference from observational data, but there are several examples of valid causal inference based on observational studies analysed using conventional approaches.  

**2) Section “Maternal genetic instrumental variables are not associated with potential confounders of the pregnancy exposure- offspring outcome association”**

 a. “Therefore, we recommend testing associations between maternal variants and observed potential confounders with and without adjustment for offspring (and paternal) genetic variants when such adjustments are used in the main MR analyses”.  We have some difficulties in understanding the practical implications of this recommendation. It could be useful to add the offspring genetic variants in the DAG reported in Figure 1 to understand what would be the impact of this adjustment on the IV – potential confounders associations. Furthermore, it would be good to clarify the concept of “testing” in this context: is it a statistical test, a judgment on the magnitude of the associations, is it at an univariable or multivariable level?

**3) Section " Maternal genetic instrumental variables are only related to the offspring outcome through their relationship to the maternal pregnancy exposure (the exclusion restriction assumption)"**

 a. We wonder if the offspring genetic variants could also affect the maternal exposures during pregnancy. It is probably unlikely, but not impossible. This would have implications for the scenario described in Supplementary File 1.

 b. Figure 2 and the last two sentences on page 5  “Thus, it is rarely possible to be able to deal with this potential new bias by adjusting for paternal (as well as offspring) genetic variants…”.  Cohorts could have information on paternal characteristics e.g. paternal BMI, paternal physical activity, smoking and other phenotypes. Adjustment for paternal phenotypes could block the path B1-->B2àB3 (even if the paternal phenotype would then become a collider).

 c. In our opinion, maternal postnatal exposures are potent potential sources of bias, whenever these exposures act on the offspring outcome through various pathways. For example maternal obesity might act on the offspring through different pathways involving diet, physical activity, sedentary behaviours, etc.

 A similar comment regards Figure 3 and page 7 – second paragraph – maternal exposure of interest as behaviour “visible” to their offspring and last sentence of the same paragraph (“less visible” exposures). We would not define these postnatal exposures as “visible” or “less visible”. In Figure 3 maternal alcohol consumption might affect offspring cognitive function or other outcomes by mechanisms other than “imitating”. In this specific case mothers with such exposures might have different child-care practice or create diverse environment where child grows that could influence later cognitive function. Maybe “manifest” and “not manifest” would work better [for example we used this terminology in Richiardi L *et al.* 2013]

**4) Section "Testing effects using maternal non-transmitted genetic variants ."**

 a. We should admit that we have not fully understood the inherent assumptions of the analysis restricted to the non-transmitted allele. For example, wouldn’t this imply an inherent conditioning on the offspring genetic variants?

**5) Supplementary file 1**

 a. “If offspring BMI is assessed in adulthood then this association is likely to be the case, as the BMI variants will relate to BMI with the same magnitude in adult mothers and their offspring when BMI is assessed in adulthood”. It should be considered however that the maternal exposure is assessed in pregnancy, while the offspring is not. Thus the strength of the IV might be different even if the assessment is made at the same age (in general, the assumption of identical effects is very stringent and it is easy to come up with examples in which this assumption is violated).

**6) Illustrative example using real data**

 a. If the standardization of the BMI is not necessary for the application of the method, we would suggest using the actual BMI in the analysis instead of BMI SDs (at least it would be good to report the magnitude of 1 SD BMI, if not already shown in the manuscript).

 b. Maybe it is out of the scope of this manuscript, but it would be interesting to discuss the implications of a possible baseline selection related with pre-pregnancy BMI in this cohort (e.g. obese mothers were more -or less- likely to participate in the study). Wouldn’t this be a source of violation of the exclusion restriction assumption (and a possible explanation for the association between the genetic score and SES and parity?)

 c. “For both BMI and FMI there was statistical evidence that the effects that both of our main MR methods were statistically inconsistent with the association from multivariable regression analyses (all p-values < 0.03)”. It seems that something is missing in this sentence.

 d. Page 11: “In the simulation analyses we examined results assuming a true null result and also a true positive effect of 0.2SD change in the offspring BMI/GFMI per 1SD change in maternal pregnancy BMI” Is this effect supported by the literature? Note that the note in Table 2 reports an assumed true result of 0.1 SD , wile the text reports 0.2 SD.

We have read this submission. We believe that we have an appropriate level of expertise to confirm that it is of an acceptable scientific standard.

## References

[1] RichiardiLPizziCPearceN: Commentary: Representativeness is usually not necessary and often should be avoided. *Int J Epidemiol*.2013;42(4) : 10.1093/ije/dyt103 1018-22 10.1093/ije/dyt103 24062290

# Peer review: Referee response for version 1

**DOI:** 10.21956/wellcomeopenres.11387.r20273

**Published:** 2017-03-20

**Authors:** C. Mary Schooling

**Affiliations:** 1 School of Public Health, Li Ka Shing Faculty of Medicine, The University of Hong Kong, Pokfulam, Hong Kong

This is a well-written article clearly addressing the specific, topic which also gives a nice explanation of some the newer Mendelian randomization (MR) concepts and techniques.

 The paper considers 3 major issues that could arise when using MR to estimate the effect of maternal attributes on offspring outcomes, shown in Figures 1, 2 and 3. It would be very helpful if at the beginning of the section "Mendelian randomization assumptions and assessing causal intrauterine effects" these three different issues were introduced, before discussing Figure 1.

 In Figure 1 it seems a bit redundant to write the IV assumptions because they can be read off the directed acyclic graph. However it may help some readers.

 In everyday life it is generally understood that causes, when relevant, act consistently. It seems unnecessary to test that genetic variants act the same in pregnant women as at other times. Instead, it might only be necessary to test that genetic variants act consistently if there was some reason to think otherwise. 

 The explanation for the result that in some cases the unadjusted MR result can be corrected by subtracting 0.5 (pages 7/8) needs to be included in the text, at least as an intuitive explanation, not just given in a Supplementary File. 

 From the regularity of the patterns in Table 1 it looks like there is an analytic solution for the simulation. If such an analytic solution could be found and added to the paper it would be very helpful and make the paper more generally applicable.

 Does weak instrument bias also likely inflate the estimate? A weak instrument might mean the denominator of a ratio is likely to be smaller than it should be.

I have read this submission. I believe that I have an appropriate level of expertise to confirm that it is of an acceptable scientific standard.

# Peer review: Referee response for version 1

**DOI:** 10.21956/wellcomeopenres.11387.r20886

**Published:** 2017-03-13

**Authors:** Brandon L. Pierce

**Affiliations:** 1 Departments of Public Health Sciences , Comprehensive Cancer Center, University of Chicago, Chicago, IL, USA

This is a well-written and important paper, as it considers a special case of MR when maternal genetic variation is use as IV to test to effect of maternal/intrauterine exposures on offspring outcomes. There are specific challenges that arise in such studies, as the IV used for the maternal exposure is likely correlated with genetic factors in the offspring which could affect offspring outcomes (violating the exclusion restriction). Also, adjusting for offspring genotype could introduce collider bias. This paper describes these (and other) potential biases and recommends approaches for avoiding these biases. No major changes suggested, but I have a few comments that may help improve the paper:

- It seems that the real data example used here was very recently published by the authors in PLOS Medicine. Assuming this is true, I suggest acknowledging this somewhere with a comment on how the data analysis presented here is different or expands on the published work, or if it is essentially the same.   
- The authors state that assortative mating could result in bias. Is this an issue specific to maternal exposures? Or a more general problem for any MR study?
- Figure 3 describes violations of the ER due to pre- and post-natal pathways. Based on this example (alcohol consumption), it seems reasonable to suspect that if one variant violates the exclusion restriction then all variants use as IV(s) would potentially violate the exclusion restriction through the same pathway. So in this case, would MR-egger and median methods might not be useful (as suggested they would be on page 9)? If not, perhaps an alternative example would better illustrate the point you’re trying to make.
- The authors state that maternal exposures will typically be studied in the one-sample setting (page 10), which can be problematic for MR-egger. This was counter-intuitive to me because information on the IV-maternal exposure association can be obtained from external data sets and use in a two-sample MR analysis. My confusion was later cleared up in the discussion, where the authors mention that this 2-sample approach is possible. In my opinion, it is not necessary to wait until the discussion to make this point. Furthermore, you may want to reconsider the claim that maternal exposures will generally be studies in the one-sample MR setting. 

 

 Minor comments:

- Commas placement not correct: “The simulation studies suggest that whether we assume a null, or positive, association the MR method …”
- “Pre/post-pregnancy” is used in text, while “pre/post-natal” is used in Figure 3. Better to be consistent. 
- Title and abstract are reasonable, but consider a more concise title. One suggestion: “Recommendations for avoiding bias in Mendelian randomization studies of maternal exposures and offspring outcomes”.

I have read this submission. I believe that I have an appropriate level of expertise to confirm that it is of an acceptable scientific standard.
